# Supplementary material for: Improved Electrochemical Performance of Li-Rich Cathode Materials via Spinel Li2MoO4 Coating
Source: Materials (Basel). 2023 Aug 17;16(16):5655. doi: 10.3390/ma16165655 (PMC10456268; doi:10.3390/ma16165655)
Supplement: Supplementary file 1 [file materials-16-05655-s001.zip › materials-2510823-supplementary.pdf]

Supporting Information

Improved electrochemical performance of Li-Rich Cathode Materials via Spinel Li_2MoO_4 Coating

Shuhao Zhang [1] Yun Ye [1,2,] Zhaoxiong Chen [1] Qinghao Lai [1] Tie Liu [3] Qiang Wang [3] Shuang Yuan [1,*]

1 School of Metallurgy, Northeastern University, Shenyang 110819, China

2 Key Laboratory for Ecological Metallurgy of Multimetallic Mineral, Ministry of Education, Northeastern University, Shenyang 110819, China

3 Key Laboratory of Electromagnetic Processing of Materials, Ministry of Education, Northeastern University, Shenyang 110819, China

* Correspondence: yuans@smm.neu.edu.cn; Tel.: 86-024-83681171

Table S1. Composition of cational elements in sample Pristine, 1wt%, 3wt% and 5wt% as measured by ICP-OES.

Samples	Sampling quality(g)	Constant volume(mL)	Measured element	Instrument reading(mg/L)	Converted content (mg/kg)	Sample element content(%)
Pristine	0.0548	25	Li	2.011	91734.5	9.17%
	0.0548	25	Li	2.011	91723.9	9.17%
	0.0548	25	Mn	7.587	346126.4	34.61%
	0.0548	25	Mn	7.584	345969.7	34.60%
	0.0548	25	Co	1.978	90258.6	9.03%
	0.0548	25	Co	1.979	90285.8	9.03%
	0.0548	25	Ni	1.701	77585.9	7.76%
	0.0548	25	Ni	1.710	78006.2	7.80%
1wt%	0.0644	25	Mo	1.352	5248.0	0.52%
	0.0644	25	Mo	1.358	5271.7	0.53%
3wt%	0.0557	25	Mo	3.430	15394.1	1.54%
	0.0557	25	Mo	3.429	15389.2	1.54%
5wt%	0.0486	25	Mo	5.057	26011.3	2.60%
	0.0486	25	Mo	5.061	26032.0	2.60%

Table S2. ICP-OES results of the obtained samples.

	Target material		ICP data
Pristine	$Li_{1.2}Mn_{0.54}Co_{0.13}Ni_{0.13}O_2$		$Li_{1.127}Mn_{0.537}Co_{0.131}Ni_{0.113}O_2$
Mass proportion of Li_2MoO_4 coating layer			
Designed	1wt%	3wt%	5wt%
ICP data	0.95wt%	2.79wt%	4.71wt%

The design value is the mass fraction of $(NH_4)_2MoO_4$ relative to Li-rich materials, while the experimental value is the mass fraction of Li_2MoO_4 relative to the overall sample.

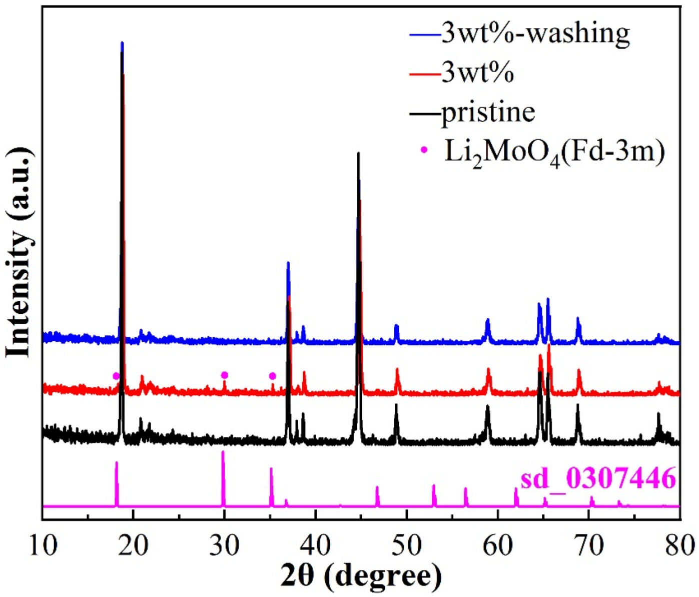

Figure S1. XRD spectrum analysis of spinel Li_2MoO_4 phase.

The peaks observed near 18, 30, and 35° are completely matched with the peaks of pure Li_2MoO_4 with spinel Fd-3m crystal structure (sd_0307446), and based on the good water solubility of Li_2MoO_4, the peak of Li_2MoO_4 disappears after washing with deionized water [1].

https://materials.springer.com/isp/crystallographic/docs/sd_0307446

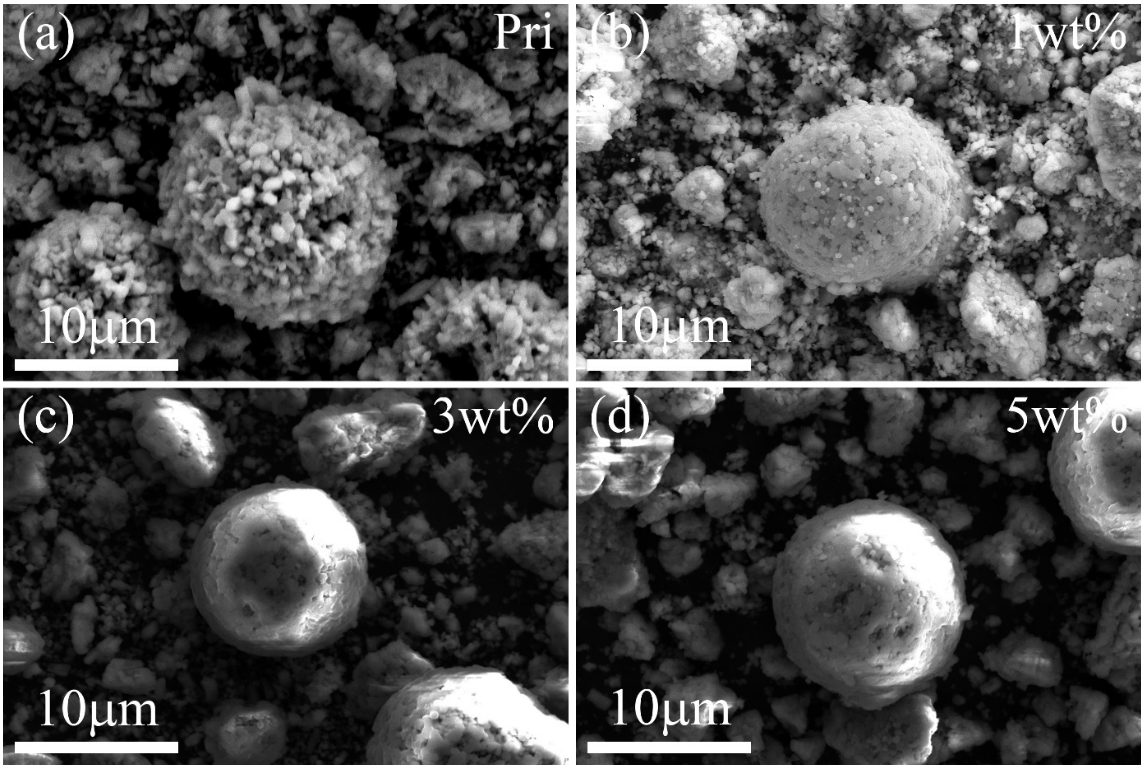

Figure S2. SEM images for (a) Pristine, (b) 1wt%, (c) 3wt%, (d) 5wt%.

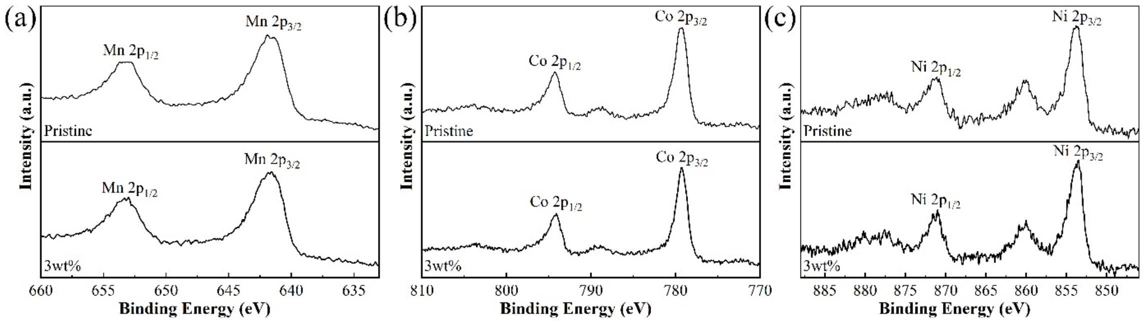

Figure S3. XPS spectra of (a) Mn 2p, (b) Co 2p, and (c) Ni 2p for Pristine and 3wt% sample.

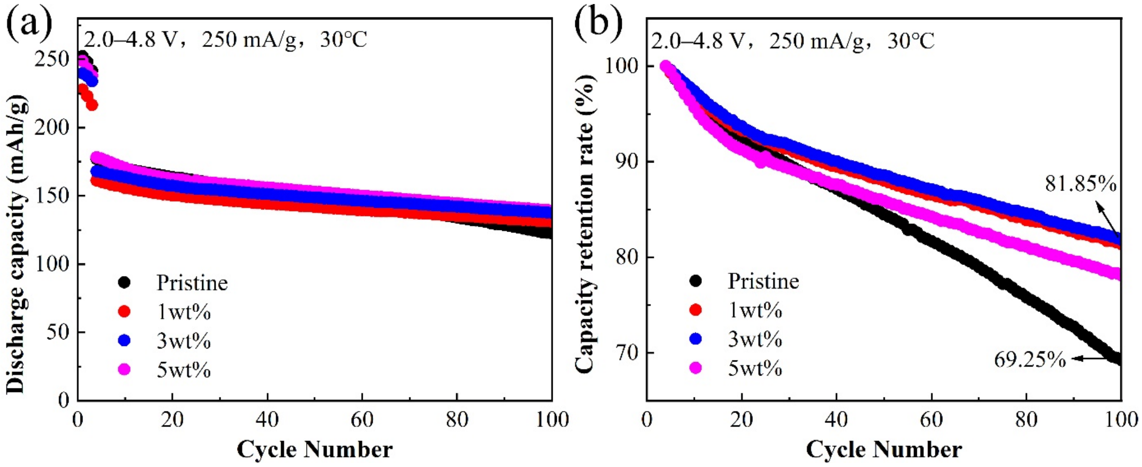

Figure S4. The cycling performance (a) and capacity retention rate (b) of four samples at 1C.

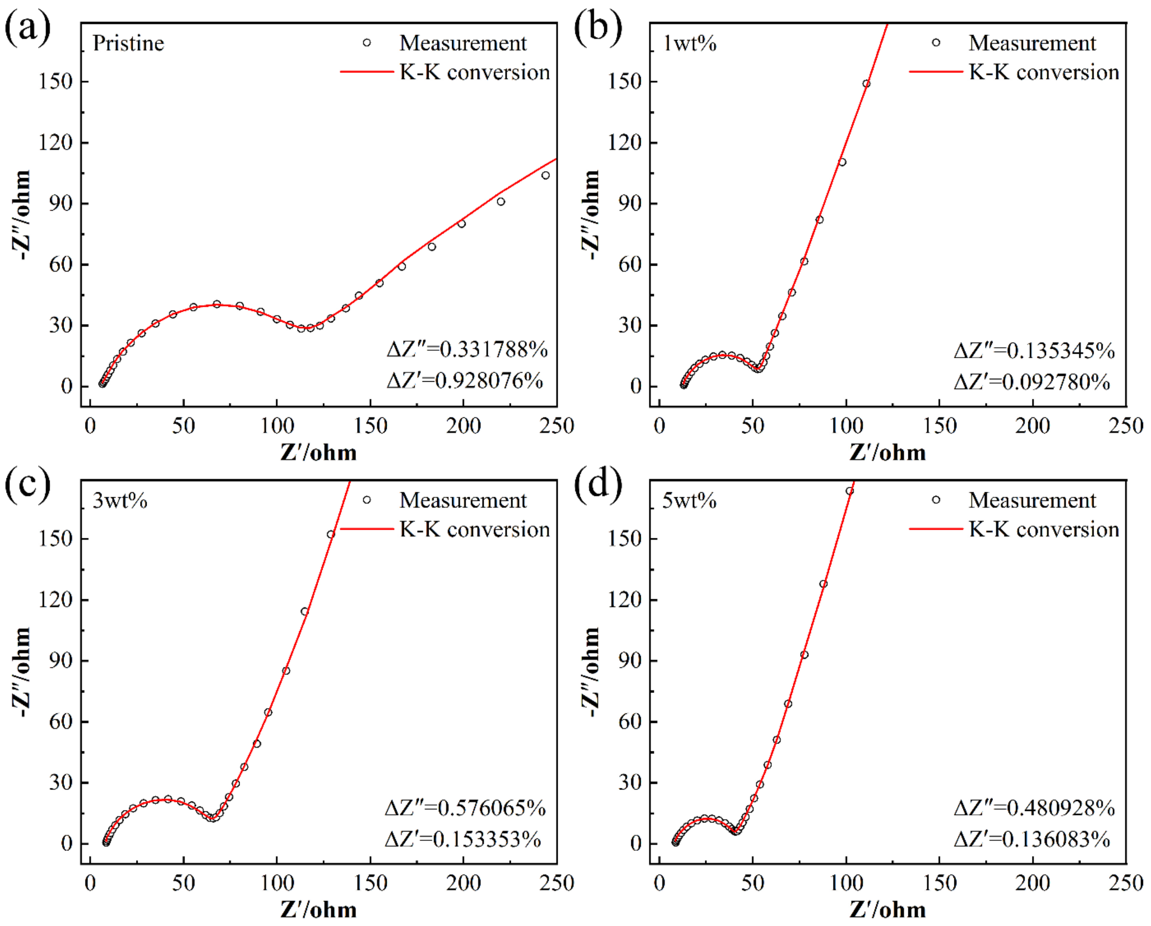

Figure S5. The Kramers-Kronig residual analysis of the four samples.

The Kramers-Kronig conversion values corresponding to the experimental measured values were obtained by ZView software, and the residual error between the measured values and the Kramers-Kronig conversion values was calculated by the following formula [2,3].

$$\Delta Z = \frac{\sum_{x=1}^{N} |Z_{ex}(x) - Z_{K-K}(x)|}{N \times |Z|_{max}}$$

In the above formula, $|Z_{ex}(x)-Z_{K-K}(x)|$ is the absolute value of the difference between the experimental measured value and the Kramers-Kronig conversion value, $|Z|_{max}$ is the maximum value of the absolute value of the experimental measured value, and N is the number of sampling points.

It can be seen from Figure S5 that the experimental measured values of EIS of the four samples are in good agreement with Kramers-Kronig conversion values, and the residual errors are all less than 1%, indicating that the EIS data satisfy Kramers-Kronig relationship and can be used to study the kinetic properties of prepared samples [3].

References

1. Zhu Z, Gao R, Waluyo I, Dong Y H, Hunt A, Lee J, Li J. Stabilized Co-Free Li-Rich Oxide Cathode Particles with An Artificial Surface Prereconstruction. *Advanced Energy Materials*, **2020**, *10*, 2001120.
2. Bastidas J M, Polo J L, Torres C L, Cano E. A study on the stability of AISI 316L stainless steel pitting corrosion through its transfer function. *Corrosion Science*, **2001**, *43*, 269-281.
3. Weiss A, Schindler S, Galbiati S, Danzer M A, Zeis R. Distribution of Relaxation Times Analysis of High-Temperature PEM Fuel Cell Impedance Spectra. *Electrochimica Acta*, **2017**, *230*, 391-398.
